# Tracking Murine Gammaherpesvirus 68 Infection of Germinal Center B Cells *In Vivo*

**DOI:** 10.1371/journal.pone.0033230

**Published:** 2012-03-13

**Authors:** Christopher M. Collins, Samuel H. Speck

**Affiliations:** 1 Emory Vaccine Center, Emory University School of Medicine, Atlanta, Georgia, United States of America; 2 Department of Microbiology and Immunology, Emory University School of Medicine, Atlanta, Georgia, United States of America; Lisbon University, Portugal

## Abstract

Infection of mice with murine gammaherpesvirus 68 (MHV68) provides a tractable small animal model to study various aspects of persistent gammaherpesvirus infection. We have previously utilized a transgenic MHV68 that expresses enhanced yellow fluorescent protein (EYFP) to identify infected cells. While this recombinant MHV68 has been useful for identifying infected cell populations by flow cytometry, it has been suboptimal for identification of infected cells in tissue sections due to the high solubility of EYFP. Efficient detection of EYFP expressed from the MHV68 genome in tissue sections requires fixation of whole organs prior to sectioning, which frequently leads to over-fixation of some cellular antigens precluding their detection. To circumvent this issue, we describe the generation and characterization of a transgenic MHV68 harboring a fusion gene composed of the EYFP coding sequence fused to the histone H2B open reading frame. Because the H2bYFP fusion protein is tightly bound in nucleosomes in the nucleus it does not freely diffuse out of unfixed tissue sections, and thus eliminates the need for tissue fixation. We have used the MHV68-H2bYFP recombinant virus to assess the location and distribution of virus infected B cells in germinal centers during the peak of MHV68 latency *in vivo*. These analyses show that the physical location of distinct populations of infected germinal center B cells correlates well with their surface phenotype. Furthermore, analysis of the distribution of virus infection within germinal center B cell populations revealed that ca. 70% of MHV68 infected GC B cells are rapidly dividing centroblasts, while ca. 20% have a clear centrocyte phenotype. Finally, we have shown that marking of infected cells with MHV68-H2bYFP is extended long after the onset of latency – which should facilitate studies to track MHV68 latently infected cells at late times post-infection.

## Introduction

Murine gammaherpesvirus 68 (MH68) infection of mice is a small animal model that has been useful in addressing basic aspects of gammaherpesvirus pathogenesis. MHV68 is related to the human gammaherpesviruses Epstein-Barr virus (EBV) and human herpesvirus 8 (HHV-8; also known as Kaposi's sarcoma associated herpesvirus [KSHV]) – both of which exhibit a very narrow host tropism and thus have been difficult to study in vivo [1]. As such, infection of mice with MHV68 allows the study of gammaherpesvirus pathogenesis during the establishment of infection that cannot be addressed for the human viruses. However, one difficulty in studying MHV68 infection is the low frequency of virally infected cells. At the peak of latency in the spleen, the frequency of infected cells is less than 1% of total splenocytes [2]–[4]. Further complicating the analysis of latently infected cells is that there is minimal viral gene transcription. In an effort to identify infected cells *in vivo*, we previously constructed a transgenic virus, MHV68-YFP, that expresses the enhanced yellow fluorescent protein (EYFP) from an expression cassette cloned into the intergenic region between orfs 27 and 29b [4]. This virus has proven to be useful for: (i) identifying infected cells early during the establishment of latency [4], (ii) tracking changes in infected cell populations when specific viral genes are knocked out [5], (iii) tracking populations of infected cells in knockout strains of mice [6], and (iv) monitoring infection *in vitro* [7], [8].

Although MHV68-YFP has been useful in identifying infected cells during the early establishment of latency, one limitation of this recombinant virus is that it is not very useful for identifying infected cells in tissue sections based on EYFP expression. This appears to be directly related to the high solubility of fluorescent proteins such as EYFP. Thus, when trying to identify infected cells in tissue sections based on EYFP expression extensive fixation is required using reagents, such as paraformaldehyde, that cross-link EYFP to cellular proteins to preserve the EYFP signal. In the absence of cross-linking, EYFP freely diffuses out of tissue sections. While small organs such as lymph nodes do not require prolonged fixation, larger organs such as the spleen require extensive fixation. This often results in over-fixation of cellular antigens, resulting in signal loss. Furthermore, this prolonged fixation often makes spleens rigid and difficult to section. These issues have previously been resolved by perfusion of whole animals with paraformaldehyde [9] which preserves detection of EYFP in tissue section, but is gentle enough to preserve detection of cellular antigens. However, this results in fixation of the entire spleen and precludes recovery of a portion of unfixed spleen for experimental analyses requiring single cell suspensions (e.g., for flow cytometry and limiting dilution analyses). Because of these issues, we pursued development of a strategy to detect infected cells in tissue sections without the complications that arise from the need for fixation.

A further limitation of the MHV68-YFP recombinant virus is that EYFP expression quickly wanes after the onset of latency [4]. This is most likely due to epigenetic modification of the region of the MHV68 genome that the EYFP expression cassette is cloned into. Notably, this region of the viral genome encodes genes that are only expressed during the lytic cycle and are not expressed during latency [10], [11]. Various strategies to extend transgene expression, including the use of other viral promoters (SV40 early promoter), cellular house-keeping gene promoters (ubiquitin C and e1f-alpha promoters), and herpesvirus promoters that are known to be active during latency (KSHV PAN promoter), have to date been universally unsuccessful in marking latently infected cells *in vivo* (Collins, Morales & Speck, unpublished data). Additionally, cloning an EYFP expression cassette into a region of the viral genome that is transcriptionally active during the early stages of MHV68 latency also failed to mark latently infected cell populations (Collins & Speck, unpublished data).

Fusion proteins consisting of histone H2B and fluorescent proteins have been used to label nuclei for tracking cells, and in studies on cell division and replication history [12]–[14]. These fusion proteins are incorporated into nucleosomes, allowing direct visualization of the chromatin in living tissues. Because these fusions are bound in nucleosomes in the nucleus, we hypothesized that this would restrict the mobility of EYFP, thereby eliminating the need to fix spleen sections. Additionally, it has been shown that a histone H2B-GFP fusion remains stable for many weeks *in vivo* in populations of slowly cycling cells [12]. Because MHV-68 has been shown to establish long term latency in memory B cells, a population of cells that divide only sporadically, we reasoned that expression of the H2bYFP fusion protein would extend marking of latently infected cells long after transcription of the H2bYFP transgene from the viral genome had been shut down. Here we show that a recombinant virus, MHV68-H2bYFP, which expresses a histone H2bYFP fusion protein can be used to detect infected cells in unfixed spleen sections. Furthermore, we show that the physical location of infected cells in these sections correlates with the surface phenotype as determined by flow cytometry. Additionally, we show that MHV68-H2bYFP is able to efficiently mark cells at late times post-infection.

## Results and Discussion

### Construction of recombinant MHV68 expressing an H2bYFP fusion protein

**Figure 1:**
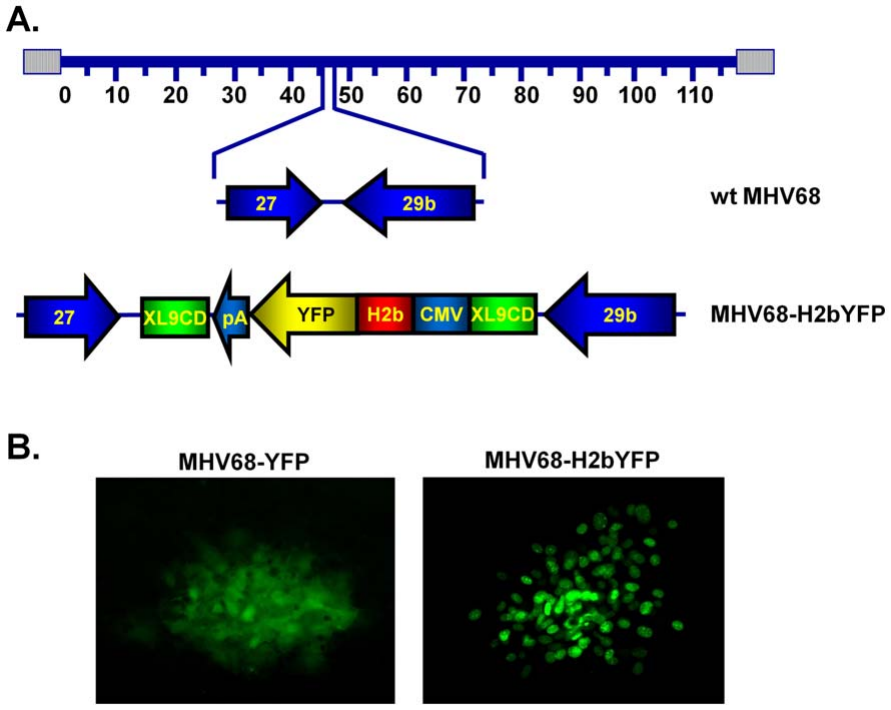
Construction of MHV68-H2bYFP. A) An expression cassette that expresses a histone H2bYFP fusion protein was cloned into the intergenic region between ORFs 27 and 29b. The cassette was flanked by chromatin insulators (XL9CD) as previously described [4] B). Representative plaques formed on NIH3T12 cells infected with either MHV68-YFP (left panel) or MHV68-H2bYFP (right panel) at 48 hours post-infection. In cells infected with MHV68-YFP, YFP has a diffuse localization that can be detected throughout infected cells, whereas YFP signal in cells infected with MHV68-H2bYFP is restricted to the nucleus.

To create a transgenic virus that will allow detection of infected cells in unfixed tissue sections, we cloned an expression cassette that expresses a fusion protein consisting of histone H2B and EYFP into the region between orfs 27 and 29b of the MHV68 genome (MHV68-H2bYFP) (fig. 1A). We have previously cloned expression cassettes into this region of the viral genome with no detectable effect on the ability of the virus to replicate or establish persistent infection [4], [15]. Correct insertion of the H2bYFP fusion gene was confirmed by PCR with primers specific for the fusion gene, as well as gel electrophoresis of purified DNA digested with diagnostic restriction enzymes. Figure 1B shows fluorescent protein expression in viral plaques formed on virus infected NIH3T12 cells at 48 hours post-infection with either MHV68-YFP (left-hand panel) or MHV68-H2bYFP (right-hand panel). In cells infected with MHV68-YFP, EYFP is diffusely distributed throughout both the cytoplasm and nucleus. However, in cells infected with MHV68-H2bYFP the distribution of the h2bYFP fusion protein is restricted to the nucleus.

### Expression of H2bYFP does not alter latency

**Figure 2:**
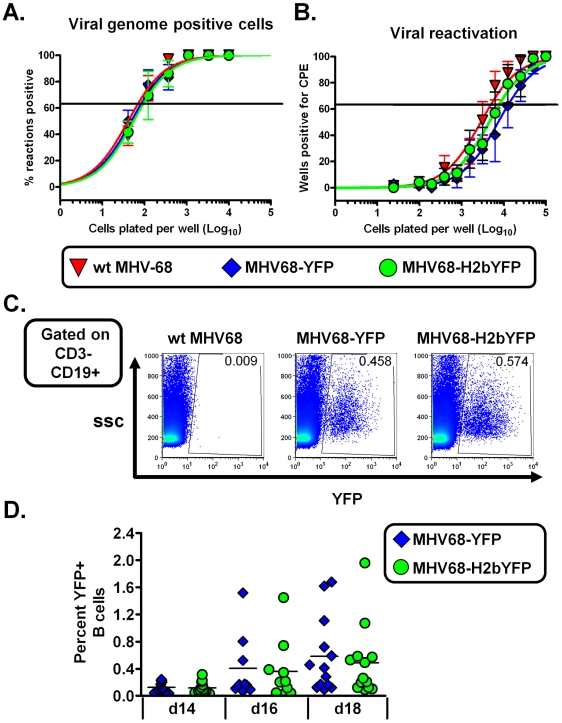
Expression of the H2bYFP fusion does not alter the ability of MHV68-H2bYFP to establish infection. A) Limiting dilution PCR analysis to determine the frequency of viral genome positive splenocytes from mice infected with 1,000 pfu of the indicated virus and harvested at 16 days post-infection. B) Limiting dilution analysis to determine the frequency of splenocytes capable of reactivating virus. Serial dilutions of splenocytes were plated on MEF indicator monolayers, and the presence of reactivating virus was determined by the presence of cytopathic effect (CPE). C) Representative flow plots showing the gating strategy to determine the percentage of EYFP and H2bYFP positive B cells. D) Quantitation of the percentage of EYFP and H2bYFP positive B cells at the indicated days post-infection from mice infected with the 1,000 pfu of the indicated virus. Results shown in panels A and B are from 3 individual experiments with 3 to 5 mice per group. Error bars represent the standard error of the mean. Each symbol in panel D represents a single mouse, and the horizontal bars represent the mean. Results are from 3 independent experiments with 3–5 mice per group.

To ensure that expression of the H2bYFP fusion protein had no impact on the ability of the virus to establish latency, mice were infected intranasally with 1,000 pfu of virus and splenocytes were harvested at 16 days post-infection. Limiting dilution PCR analyses showed that the frequency of viral genome positive splenocytes from mice infected with MHV68-H2bFYP was similar to both wild type virus and MHV68-YFP (Fig. 2A). Approximately 1 in 95 splenocytes from mice infected with MHV68-H2bYFP were viral genome positive compared to 1 in 80 splenocytes from wild type virus infected mice and 1 in 70 splenocytes from mice infected with MHV68-YFP.

The ability to reactivate from latency was also determined by a limiting dilution *ex vivo* reactivation assay (Fig. 2B). As expected, there was no significant difference in the ability to reactivate from latency between MHV68-H2bYFP and either wild type virus or MHV68-YFP. Approximately 1 in 4,900 splenocytes were capable of reactivating virus from mice infected with wild type virus compared to 1 in 12,500 splenocytes from mice infected with MHV68-YFP and 1 in 7,800 splenocytes from mice infected with MHV68-H2bYFP. The observed differences are within the normal variability that we have previously observed using this assay (as we have previously documented, and as shown in Fig. 2D, significant mouse to mouse variation in the frequency of virus infected splenoctyes is observed – which necessitates pooling splenocytes from 4–5 mice for assessment of latency) [4].

To assess whether expression of the H2bYFP fusion protein had any impact on the ability to mark cells, the percentage of EYFP positive B cells from mice infected with MHV8-H2bYFP was compared to that of splenocytes from mice infected with MHV68-YFP (Fig. 2 panels C & D). Notably, there was no difference in the ability of MHV68-H2bYFP to mark B cells compared to MHV68-YFP. Although there was some individual variation, there was no significant difference in the average number of EYFP positive cells from mice infected with MHV68-H2bYFP compared to MHV68-YFP. At day 14 post-infection, the average number of EYFP positive splenic B cells from mice infected with MHV68-YFP was 0.125% compared to 0.116% from mice infected with MHV68-H2bYFP. By day 18 post-infection the percentage of EYFP positive B cells had risen to 0.58% and 0.49% for MHV68-YFP and MHV68-H2bYFP, respectively. Taken together, this data shows that expression of the H2bYFP fusion protein does not appear to have any impact on the ability of MHV68 to establish infection, reactivate from latency or mark B cells.

### Analysis of MHV68 infection of germinal center B cells

**Figure 3:**
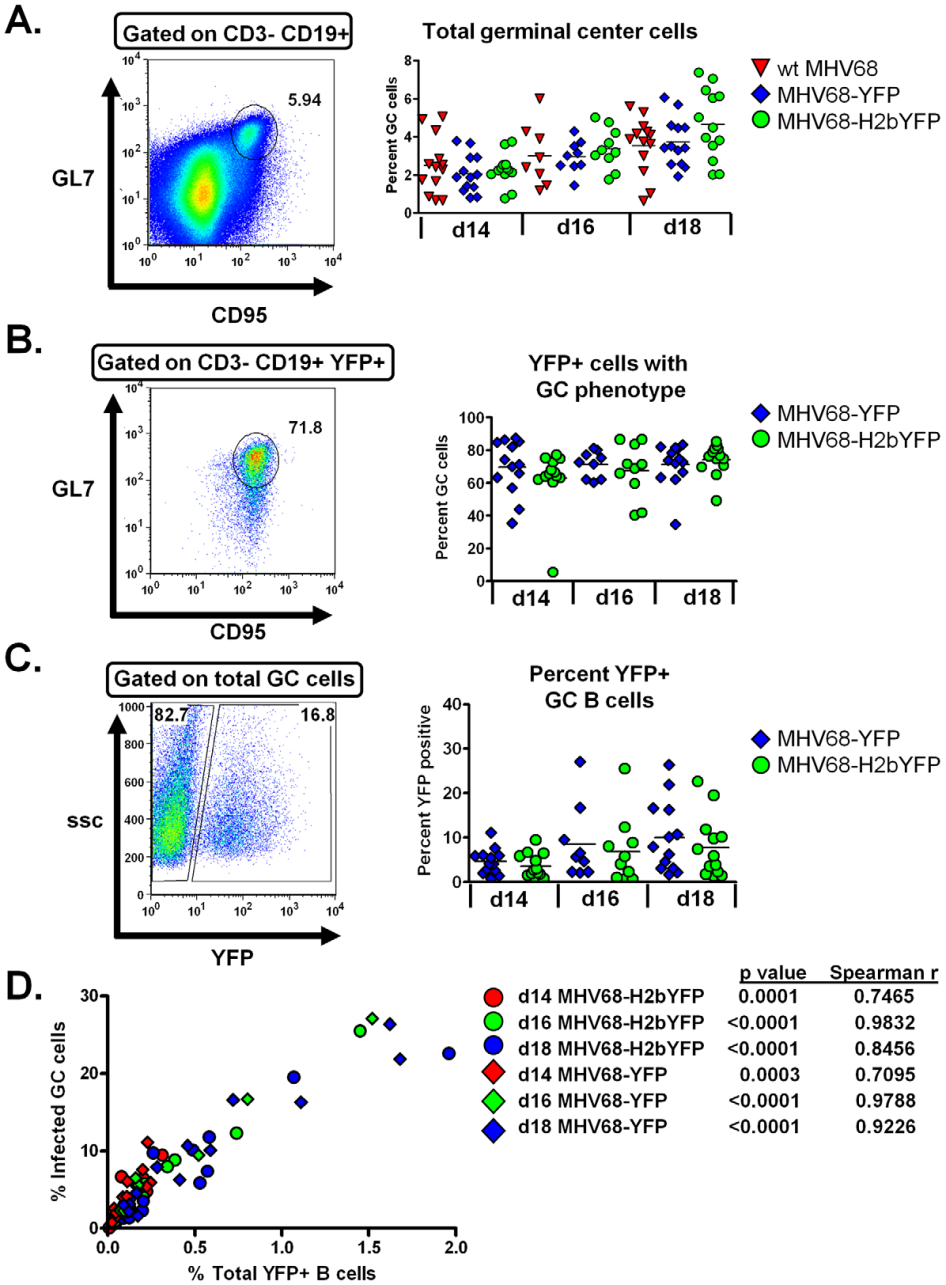
Expression of H2bYFP does not alter germinal center dynamics of MHV68-H2bYFP. Mice were infected with 1,000 pfu of the indicated virus and spleens were harvested at the indicated day post-infection. A) Total germinal center B cell analysis. A representative flow plot showing the gating strategy to identify total germinal center population is shown in the left panel. B cells were defined as CD19^+^ CD95^HI^ GL7^HI^. Quantitation of the percentage of B cells with a germinal center phenotype is shown in the right panel. B) Percentage of EYFP and h2bYFP positive cells that have a germinal center phenotype. Representative flow plot showing the gating strategy used to identify cells with a germinal center phenotype in the EYFP and h2bYFP positive fraction is shown in the left panel. The percentage of EYFP and H2bYFP positive cells that have a germinal center phenotype is shown in the right panel. C) Percentage of germinal center cells that are infected. A representative flow plot showing the gating strategy to identify the percentage germinal center cells that are infected based on YFP expression (left panel). The percentage of germinal center cells that were infected is shown in the right panel. D) Correlation between the percentage of EYFP and H2bYFP positive B cells and the percentage of EYFP and H2bYFP positive germinal center cells. In the right panels of A, B and C, the horizontal bar represents the mean.

Analysis of the total germinal center B cell populations showed that there was no significant difference in the average number of GC B cells at either days 14, 16 or 18 post-infection in mice infected with either wild type virus, MHV68-YFP or MHV68-H2bYFP (Fig. 3A). The percentage of germinal center B cells rose slightly from ca. 2% at day 14 to ca. 4% by day 18 post-infection. Although the overall percentage of B cells exhibiting a germinal center phenotype increased from days 14 to 18 post-infection, the percentage of EYFP positive cells exhibiting a germinal center phenotype remained relatively constant at ca. 70% at each time point (Fig. 3B). Importantly, expression of the H2bYFP fusion protein had no impact on the ability of MHV68-H2bYFP to gain access to the germinal center population. There was also no significant difference in the overall percentage of germinal center B cells that were infected based on EYFP expression from mice infected with MHV68-H2bYFP compared to MHV68-YFP (Fig. 3C). However, there was some individual variation in the percentage of infected germinal center cells, and in some animals nearly 30% of their germinal center B cells were EYFP positive. To determine if the increased percentage of infected germinal center cells correlated with the overall level of infection, the percentage of EYFP positive germinal center B cells was plotted against the percentage of total EYFP positive B cells (as shown in Fig. 2D). Notably, this analyses revealed a very strong correlation between the overall levels of infection as measured by the percent EYFP+ splenocytes, and the percentage of germinal center B cells that were infected (Fig. 3D). Animals that had the highest percentage of infected cells also had the largest proportion of infected germinal center B cells. Conversely, in animals that had relatively few EYFP positive B cells, infected germinal center cells made up a small percentage of the total germinal center B cell population.

### Detection of H2bYFP positive cells in tissue sections

**Figure 4:**
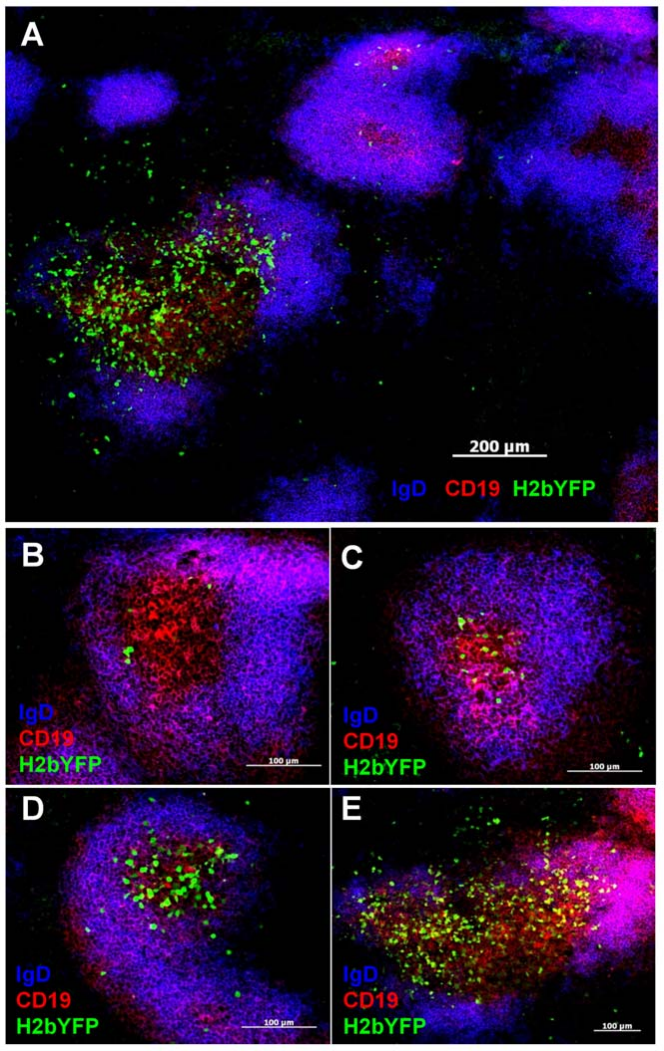
Detection of H2bYFP positive cells in unfixed spleen sections. A) Representative spleen section from a mouse infected with 1,000 pfu of MHV68-H2bYFP and harvested at day 16 post-infection. B–E) H2bYFP positive germinal centers demonstrating the variability in the number of infected cells per germinal center. Images were collected at a magnification of 100× in A, 320× in B–D, and 200× in E.

To assess H2bYFP expression in unfixed spleen sections, mice were infected intranasally with 1,000 pfu of MHV68-H2bYFP. At day 16 post-infection, spleens were harvested and sections were analyzed for H2bYFP expression. While we have encountered significant difficulties detecting EYFP expression in spleens from mice infected with MHV68-YFP (not shown), we could easily detect H2bYFP expression in spleen sections from mice infected with MHV68-H2bYFP (Fig. 4). Figure 4A shows a representative section co-stained with anti-CD19 to detect B cell follicles, as well as anti-IgD to delineate germinal centers within follicles that have down-regulated expression of IgD. Consistent with the phenotypic analyses summarized in figure 2B, as well as previous reports that the majority of infected splenocytes have a germinal center phenotype [3], [4], the majority of H2bYFP positive cells were found in germinal centers - although a few H2bYFP positive cells were found outside germinal centers. Both H2bYFP-positive and -negative germinal centers were observed and, similar to variation we see in the total number of EYFP positive B cells (see Fig. 2D), there was considerable variation in the number of H2bYFP positive germinal centers per section. In some animals, nearly all germinal centers contained H2bYFP-positive cells, whereas in others there were as few as 1–2 H2bYFP-positive germinal centers per section. Additionally, there was significant variation in the number of H2bYFP-positive cells per germinal center. Representative sections shown in figure 4, panels B-E, illustrate the wide range in the number of H2bYFP-positive cells per germinal center we observed.

**Figure 5:**
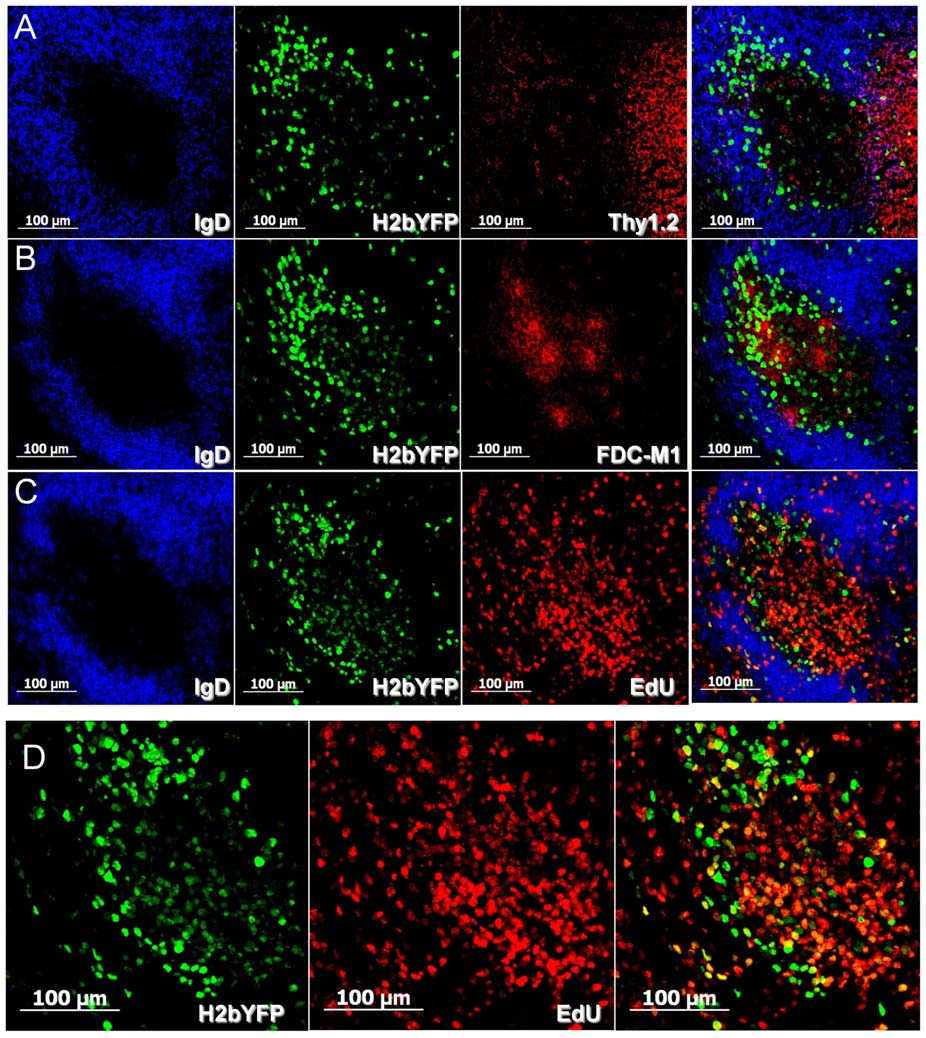
Localization of H2bYFP positive cells within germinal centers. Serial sections through a representative splenic GC recovered at day 16 post-infection, co-stained with markers to localize infected cells within specific germinal center compartments. Sections were stained with anti- IgD to delineate the mantle zone surrounding the GC and anti-GFP to detect infected cells. To detect the T cell zone, sections were co-stained with anti-Thy 1.2 (row A). The light zone is detected by the presence of follicular dendritic cells stained with anti-FDC-M1 (row B). Rapidly dividing centroblasts are found in the dark zone, and can be detected by increased incorporation of EdU (row C). Row D shows the h2bYFP and EdU panels from row C overlayed without IgD to identify proliferating H2bYFP positive cells.

To further localize H2bYFP-positive cells within specific compartments of the germinal center, spleen sections were stained with antibodies to various markers used to define specific components of germinal centers. Figure 5 shows serial sections through a representative germinal center. The sections have been stained with anti-IgD to define the mantle zone surrounding the germinal center, and anti-GFP to detect the H2bYFP fusion protein being expressed in MHV68-H2bYFP infected cells. The T cell zone, found adjacent to B cell follicles, is shown in row A and was detected by staining with anti-Thy1.2. Within the germinal center, centrocytes are found in the light zone - which forms distal to the T cell zone and can be identified by the presence of follicular dendritic cells stained with anti-FDC-M1 (row B). The dark zone contains rapidly dividing centroblasts and forms proximal to the T cell zone. These rapidly dividing cells readily incorporate EdU, which can be used to define the dark zone (row C). Notably, H2bYFP-positive cells could be detected throughout the germinal center in both the light zone and dark zone, as well as a few H2bYFP positive cells that were observed in the IgD+ mantle zone and in the T cell zone (row A). There were also H2bYFP positive cells at the border between the B cell follicle and the T cell zone, a site where initial CD4 T cell help activates B cells that then migrate into the B cell follicle to initiate the germinal center reaction (reviewed in [16]). Notably, there appeared to be two distinct populations of H2bYFP positive cells - those that expressed high levels of H2bYFP and largely localized to the light zone, and those that expressed lower levels of H2bYFP and localized in the dark zone. As shown in figure 5 row D (a higher magnification of the germinal center section shown in row C), the H2bYFP positive cells and EdU positive cells (overlayed without the staining IgD staining) allowed identification of infected cells that were proliferating based on high level EdU incorporation. In the dark zone, the majority of H2bYFP positive cells were also EdU positive, although there were a few that had no detectable EdU incorporation. In the light zone, while a few H2bYFP positive cells were EdU positive, many more were EdU negative than was observed in the dark zone. The physical location of the high versus low H2bYFP expressing cells, along with the relative level of EdU incorporation, suggested that cells expressing high levels of H2bYFP are centrocytes while those expressing lower levels of H2bYFP low expressing cells are centroblasts.

**Figure 6:**
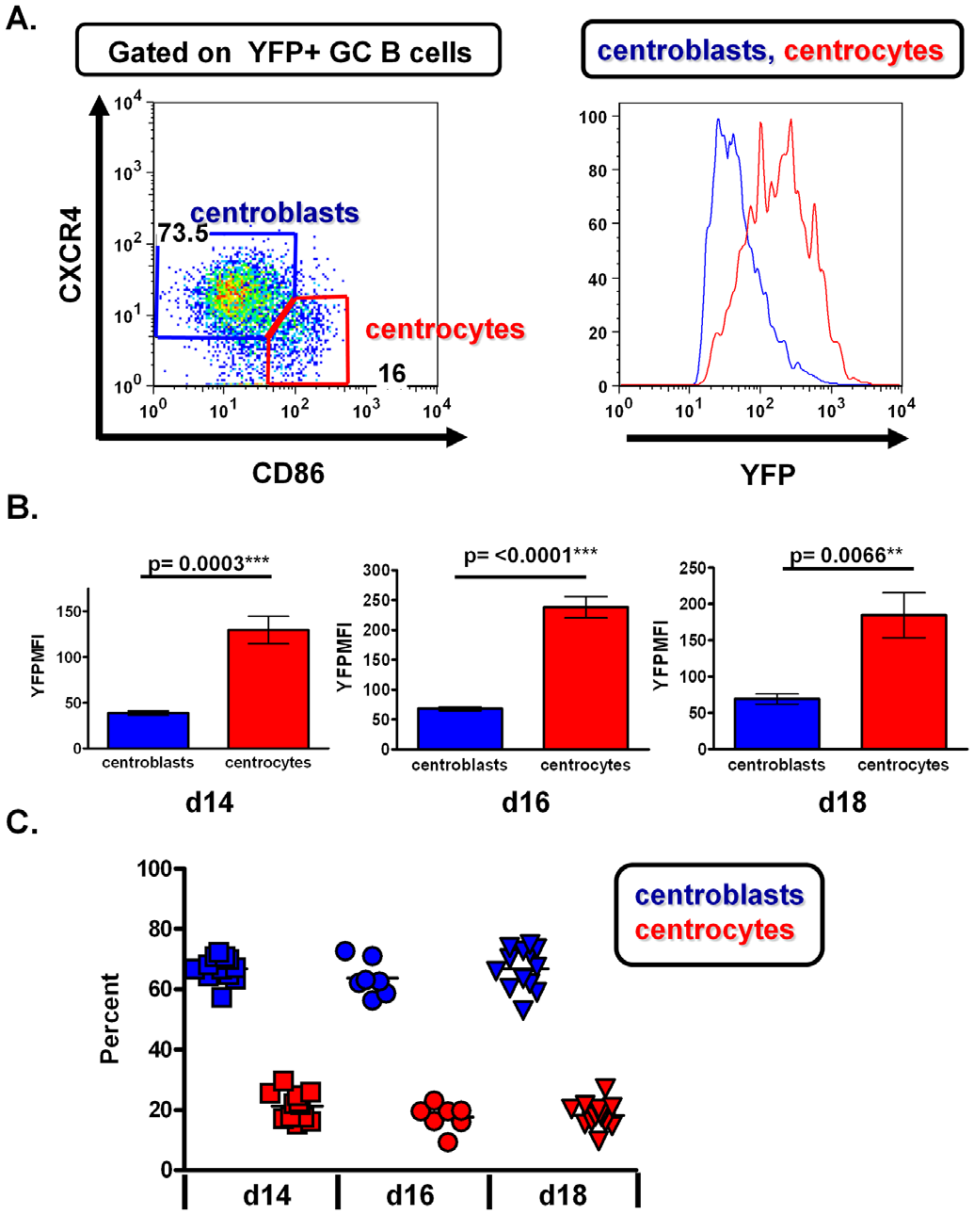
Characterization of infected centroblast and centrocyte populations. A) Gating strategy used to identify YFP positive centroblasts and centrocytes (left panel). Representative histogram showing YFP expression in centroblasts and centrocyts (right panel). B) Mean fluorescence intensities of YFP in centroblasts and centrocytes at days 14, 16 and 18 post-infection. Each graph shows one representative experiment with 4 to 5 mice per group. H2bYFP MFI was analyzed in at least 3 independent experiments at each time point with similar results. C) Quantitation of H2bYFP positive centroblast and centrocyte populations at the indicated days post-infection.

To further assess whether the difference in H2bYFP expression correlated with differences in surface phenotype, H2bYFP positive GC cells were analyzed for expression of CXCR4 and CD86. Centroblasts have been shown to be CXCR4^hi^ CD86^lo^ whereas centrocytes are CXCR4^lo^CD86^hi^ [17]. Figure 6A shows the gating strategy used to determine H2bYFP expression in these populations, along with the analysis of a representative MHV68-H2BYFP infected mouse. Compiling the data from multiple infected mice revealed a clear difference in H2bYFP expression in the centroblast and centrocyte germinal center B cell populations at days 14, 16 and 18 post-infection (Fig. 6B). Figure 6B shows the mean fluorescence intensity (MFI) of H2bYFP in splenocytes from representative experiments harvested at days 14, 16 and 18 post-infection. Furthermore, quantitation of these populations revealed that the majority of MHV68 infected germinal center cells exhibit a centroblast phenotype (ca. 3∶1 ratio of centroblasts to centrocytes) (Fig. 6C).

### Long term marking of latently infected B cells by MHV68-H2bYFP

**Figure 7:**
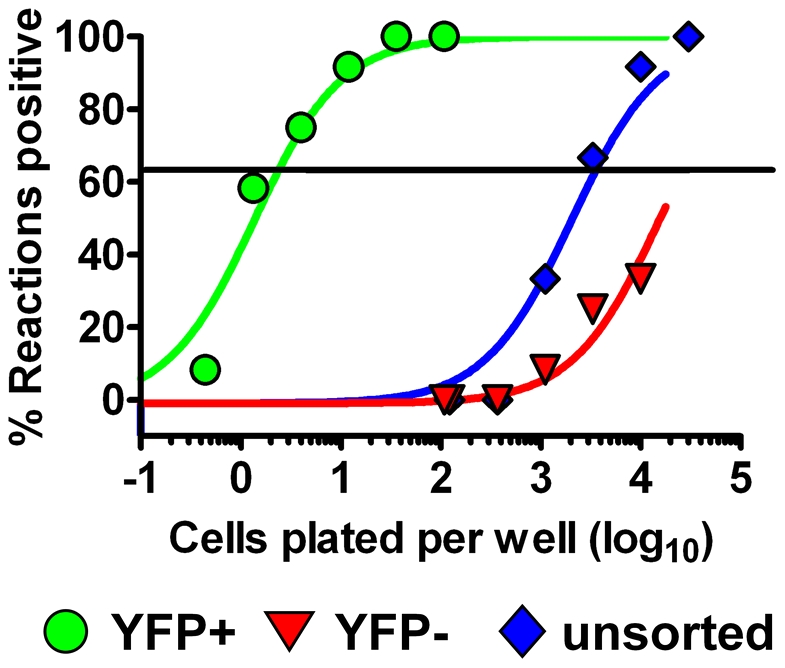
Long term marking of splenocytes infected with MHV68-H2bYFP. YFP+ and YFP− splenocytes from mice were sorted at 42 days post-infection from mice infected with 1,000 pfu of MHV68-H2bYFP. Limiting dilution PCR analysis was performed to determine the frequency of viral genome positive cells in each fraction.

Because histone H2b-GFP fusion proteins have been shown to be stably maintained in cells that cycle slowly [12], we reasoned that the H2bYFP fusion protein may extend marking of infected cells due to the fact that long term latency is maintained in memory B cells - a cell population that is largely quiescent [3]. Notably, we have previously shown that although we can sort out an EYFP positive population of infected cells from mice infected with MHV68-YFP at day 42 post-infection, there was a significant fraction of viral genome positive cells that no longer had detectable levels of YFP expression [4]. Indeed, from 2 independent analyses only ca. 6,600 and 6,800 YFP+ cells were isolated from a total of 12 pooled spleens (the theoretical number of infected cells in these samples ranged from 1.5–2.7×10^5^ cells based on limiting dilution analyses of bulk splenocytes) [4]. Thus, less than 5% of MHV68 infected cells at day 42 post-infection with the MHV68-YFP virus were recovered, suggesting inefficient EYFP expression at this late time post-infection. Here we repeated this analysis using 10 pooled spleens harvested at day 42 post-infection with MHV68-H2bYFP. We were able to purify 1.25×10^5^ H2bYFP positive splenocytes from a total of 7×10^8^ cells. This yield of H2bYFP positive cells closely correlated with the theoretical number of MHV68 infected cells (1.94×10^5^) as determined by limiting dilution analysis of the bulk splenocyte population and the total number of cells sorted (estimated recovery of ca. 65% of virus infected cells). Analysis of the H2bYFP positive cell population by limiting dilution for the presence of the MHV68 genome revealed that, within the likely error of the limiting dilution assay, virtually all recovered cells harbored viral genome (frequency of H2bYFP positive cells recovered that harbored viral genome was estimated to be 1 in 2.4) (Fig. 7). Furthermore, we observed a significant reduction in the frequency of viral genome positive cells in the H2bYFP-negative splenocyte fraction (1 in 27,000 cells) compared to the unsorted population (1 in 3,600 cells), indicating that the majority of MHV68 infected cells were H2bYFP positive. Thus, unlike the MHV68-YFP virus which efficiently marks latently infected splenocytes only at the early stages of latency, infection with the MHV68-H2bYFP virus extends the time frame of efficient marking of infected splenocytes to at least 6 weeks post-infection.

### Conclusions

In this report we describe a new strategy to mark cells infected with MHV68 that allows both long term marking, as well as detection of infected cells in unfixed tissue sections. The ability to correlate surface phenotype with physical location within tissue sections provides a new tool to study gammaherpesvirus pathogenesis. It also simplifies detection of virus infected cells since it eliminates the need for optimizing fixation of different tissue types. Furthermore, the morphology of the sections is well maintained and signal loss due to over-fixation of antigens is not an issue.

We have focused on splenic latency since the spleen is a rich source of infected cells during the early establishment of latency. However, we have also been able to detect MHV68-H2bYFP infected cells in unfixed sections of lymph nodes (C.M. Collins and S.H. Speck, unpublished data) as well as the omentum (K. S. Gray and S. H. Speck, unpublished data). It should be noted that while cell types such as macrophages and dendritic cells have been shown to harbor viral genome [2], [3], [18], [19] which are present in the spleen at the peak of viral latency, we have not been able to convincingly detect EYFP or H2bEYFP transgene expression in these cell types. To date we have only detected MHV68 infected B cells and plasma cells using either the MHV68-YFP or MHV68-H2bYFP viruses. This may reflect: (i) cell type specific activity of the HCMV IE promoter driving transgene expression; (ii) the low frequency of viral genome positive macrophages and dendritic cells present in the spleen at the peak of viral infection [2], [3]; and/or (iii) that MHV68 infection of these cells types is non-productive and does not result in significant transcription from the viral genome. Notably, previous reports have shown that the frequency of viral genome positive cells in these populations is very low [2], which would be very difficult to detect by flow cytometry above background auto-fluorescence. However, we have also failed to detect EYFP expression in peritoneal macrophages following intraperitoneal inoculation (data not shown) – an anatomical site where we have shown that a majority of the MHV68 infected cells are macrophages [19]. The latter argues that there is little or no expression of the EYFP and H2bYFP transgenes in MHV68 infected macrophages.

We do not know why there is such extreme variation in level of MHV68 infection of germinal centers. Newly activated B cells have been shown to be able to enter existing germinal centers [20], so it is possible that germinal centers with few infected cells (for example see Fig. 4B,C) may result from colonization of existing germinal centers by infected cells. In germinal centers with high levels of infection (see Fig. 4E), infected B cells may be responsible for the initiation of these germinal centers. It will be of interest to determine whether the H2bYFP positive cells in individual germinal centers with high levels of infected cells are clonal, which would indicate that they arose from single infected B cells.

Previously, cells infected with MHV-68 in lymphoid tissues have been detected by *in situ* hybridization using probes that detect the presence of vtRNAs [21]–[23]. A recent report characterizing MHV68 infection in wood mice indicated that the majority of infected cells in germinal centers were localized to the light zone based on the distribution of viral tRNAs (vtRNAs) positive cells [24]. However, as discussed above, we noted two distinct populations of germinal center B cells based on the intensity of H2bYFP expression – which reflected differences in the levels of H2bYFP expression in centroblasts vs centrocytes (see Fig. 6). Although this could reflect differences in transcription of the H2bYFP transcription in these two B cell populations, it is more likely that it reflects the high rate of proliferation of centroblasts leading to dilution of H2bYFP levels in these cells. If the latter is true, it is tempting to speculate that rapidly dividing centroblasts also express low levels of the vtRNAs and thus leading to a failure to detect MHV68 infected centroblasts by *in situ* hybridization. Indeed, it seems very likely that gammaherpesviruses have evolved to take advantage of the germinal center reaction as a means to expand the pool of latently infected B cells – some of which ultimately gain access to the memory B cell reservoir. As observed in classical T cell responses, there is a significant contraction in the frequency of MHV68 B cell latency from a peak at ca. 2 weeks post-infection (ca. 1 in 100) to the steady-state level established by 3 months post-infection (ca. 1 in 10,000) [3]. This contraction may reflect immune clearance of some population(s) of MHV68 latently infected B cells, and/or the intrinsic inefficiency in the transition of germinal center B cells to the memory B cell pool. We anticipate that the MHV68-H2bYFP virus described here will a useful tool in beginning to unravel this issue.

## Materials and Methods

### Ethics Statement

This study was carried out in strict accordance with the recommendations in the Guide for the Care and Use of Laboratory Animals of the National Institutes of Health. The protocol was approved by the Emory University Institutional Animal Care and Use Committee, and in accordance with established guidelines and policies at Emory University School of Medicine (Protocol Number: 046-2010).

### Viruses and tissue culture

Wild type MHV-68 strain WUMS (ATCC VR-1465) was used for all wild type infections. Virus propagation and titer determination was performed as previously described [25]. NIH3T12 cells were obtained from ATCC (CCL-164) and Vero-Cre cells were generous gift from Dr. David Leib [26]. Murine embryonic fibroblasts (MEFs) were generated from homogenized day 16 C57Bl6 embryos cultivated in complete DMEM as previously described [27]. NIH3T12, Vero-Cre, and murine embryonic fibroblasts (MEFs) were maintained in Dulbecco's modification of Eagle medium (DMEM) supplemented with 10% fetal bovine serum, 2 mM L-glutamine, and 100 U of penicillin and 100 mg of streptomycin per ml.

### Mice, infections and tissue harvest

Female C57BL/6J mice were purchased from Jackson Laboratory (Bar Harbor, ME) at 6 to 8 weeks of age and were infected at 8 to 12 weeks. All protocols utilized in this study were approved by the Institutional Animal Care and Use Committee of Emory University. Mice were anesthetized with isofluorane prior to infection and inoculated intranasally with 1,000 pfu of virus that was diluted in 20 µL of DMEM.

### Construction of MHV68-H2bYFP

To generate a histone h2bYFP fusion protein, the histone h2b open reading frame was PCR amplified from genomic DNA purified from splenocytes isolated from C57Bl6 mice using the primers H2B For_PstI (5′-CCGCCTGCA GACCATGCCCGAGCCTGCG-3′) and H2B_linker Rev (5′-ACTACCTCTTACACACTTGGAG CTGGTGTACTTAGTGACAGC-3′). The EYFP open reading frame was PCR amplified using the primers linker-YFP-For (5′-TGTGTAAGAGGTAGTATGGTGAGCAAGGGCGAGGAGCTG-3′) and YFP-Rev-NotI (5′-CCGCGCGGCCGCCCCCAGCTGGTTCTTTC-3′). Primers were designed to incorporate a 15 nucleotide linker at the 3′ end of the *h2b* orf and at the 5′ end on the EYFP orf. This linker sequence was then used for overlapping PCR with the primers H2B For_PstI and YFP-Rev-NotI to generate the H2bYFP fusion protein. The H2bYFP PCR product was digested with the restriction enzymes PstI and NotI and cloned into the PstI-NotI site of the previously described pCR-Blunt-Ins-CMV-YFP [4] to create pCR-Blunt-Ins-CMV-H2bYFP. The fragment spanning the hCMV IE promoter to the bovine growth hormone polyadenylation signal was then purfied by digestion of pCR-Blunt-Ins-CMV-H2bYFP with the restriction enzymes NsiI and NotI. This fragment was cloned into the NsiI NotI fragment of the previously described JE110-XL9CD CMV-YFP-R [4] to generate JE110-X-CMV-H2bYFP. The ApaI fragment from this vector was then cloned into the ApaI site of pGS284-Blunt-MCS [4] to generate pGS284-XL9CD-CMV-H2bYFP. This vector was then used for allelic exchange with wild-type MHV68 BAC in GS500, a RecA+ strain of *E. coli.*

### Limiting dilution PCR analysis

To determine the frequency of viral genome positive splenocytes, limiting dilution PCR analysis using a single copy sensitive nested PCR assay was performed as previously described [16], [17]. Briefly, serial three-fold dilutions of splenocytes were plated in 96 well plates in a background of 10^4^ uninfected NIH3T2 cells. Cells were lysed by proteinase K digestion for 6 hours at 56°C followed by heat inactivation at 95°C for 20 minutes. Samples were then subjected to 2 rounds of PCR using nested primers as previously described [18], [27]. To ensure single copy sensitivity, plasmid controls consisting of 10, 1, and 0.1 copies of the orf50 containing plasmid pBamHIN were spiked into a background of 10^4^ uninfected NIH3T12 cells. PCR products were analyzed on a 2% agarose gel.

### Limiting dilution *ex vivo* reactivation

To determine the frequency of splenocytes capable of reactivating virus, limiting dilution analysis was performed as previously described 18,27]. Splenocytes were resuspended in complete DMEM and plated onto indicator MEF monolayers in serial two-fold dilutions, beginning with 10^5^ splenocytes per well. Wells were scored for cytopathic effect 14 days after plating. To detect the presence of preformed infectious virus, parallel samples of mechanically disrupted splenocytes were also plated on MEF monolayers.

### Flow cytometry

Single cell suspensions of splenocytes were resuspended in phosphate-buffered saline containing 1% fetal bovine serum and stained for 20 minutes on ice in the dark. Antibodies used were phycoerythrin conjugated and-CD138 (BD Pharmingen), PerCP conjugated anti-CD3 (BD Pharmingen), phycoerythrin Cy7 conjugated anti-CD95 (BD Pharmingen), Pacific blue conjugated anti- B220 (Invitrogen), phycoerythrin conjugated anti-CD19 (BD Pharmingen), allophycocyanin conjugated anti-GL7 (eBioscience). Biotin conjugated anti GL7 was detected with allophycocyanin conjugated streptavidin (Invitrogen). Data were acquired on an LSRII flow cytometer (BD Biosciences) and analyzed on FloJo software (Treestar, Inc., San Carlos, CA).

For fluorescence activated cell sorting, splenocytes from 10 pooled spleens from mice infected with 1,000 pfu of MHV68-H2bYFP and harvested at 16 days post-infection were resuspended at a concentration of 1×10^8^ cells per ml in PBS containing 1% fetal bovine serum. Cells were sorted on a FACSAria (BD Biosciences) and sorted populations were immediately plated out for LD-PCR analysis.

### Tissue section preparation and immunofluoresence microscopy

Spleens from infected mice were harvested and embedded in OCT media (Sakura Finetek) by flash freezing themin chilled-isopentane (Fisher Scientific). Frozen sections were cut at a thickness of 5 mm, mounted on glass slides and stored at −80°C until needed. Sections were re-hydrated at room temperature in PBS for 10 minutes and then blocked with PBS containing 3% BSA for 20 minutes at room temperature. H2bYFP was detected in sections using a FITC conjugated anti-GFP antibody (Rockland Immunochemicals for Research). Antibodies to cellular antigens were purified anti-IgD(BD Pharmingen), purified anti-FDC-M1(BD Pharmingen), Alexa647 conjugated anti-IgD (Biolegend), phycoerythrin conjugated anti-CD19 (BD Pharmingen), and allophycocyanin conjugated anti-Thy1.2 (BD Pharmingen). Purified antibodies were detected with Alexa 546 conjugated anti-rat IgG (Invitrogen). After staining, sections were mounted with Prolong antifade reagent with DAPI (Invitrogen). Images were collected on a Zeiss Axiovert 200 M fluorescent scope using Axiovision imaging software.

### EdU labeling

To detect replicating cells in spleen sections, mice were treated with 5′-ethynyl- 2′-deoxyuridine (EdU) (Invitrogen) for 5 hours by intra-peritoneal injection of 100 mg of EdU in PBS. Cells that had incorporated EdU were detected with a Click-it EdU Alexa Fluor 555 Imaging kit (C10338) from Invitrogen according to the manufacturer's protocol with the following modifications. Sections were stained with fluorophore conjugated antibodies and subsequently fixed with 4% paraformaldehyde in PBS for 10 minutes at room temperature. After fixation, sections were permeabilized with 0.5% Triton X-100 in PBS for 15 minutes at room temperature, and EdU detection was then performed according to the manufacturer's protocol.

### Statistical analysis

All data analysis was performed using GraphPad Prism software (GraphPad Software, San Diego, CA). The frequencies of genome positive splenocytes and splenocytes capable of reactivation virus were determined by non-linear regression fit of the data where the regression line intersected 63.2%, corresponding to the frequency at which one event is predicted to be present in that population.
